# *Xenopus* Models of Cancer: Expanding the Oncologist’s Toolbox

**DOI:** 10.3389/fphys.2018.01660

**Published:** 2018-11-27

**Authors:** Laura J. A. Hardwick, Anna Philpott

**Affiliations:** ^1^Philpott Lab, Hutchison/MRC Research Centre, Department of Oncology, University of Cambridge, Cambridge, United Kingdom; ^2^Wellcome MRC Stem Cell Institute, University of Cambridge, Cambridge, United Kingdom; ^3^Peterhouse, University of Cambridge, Cambridge, United Kingdom

**Keywords:** *Xenopus*, cancer, tumour, oncogene, transgenic

## Abstract

The use of the *Xenopus* model system has provided diverse contributions to cancer research, not least because of the striking parallels between tumour pathogenesis and early embryo development. Cell cycle regulation, signalling pathways, and cell behaviours such as migration are frequently perturbed in cancers; all have been investigated using *Xenopus*, and these developmental events can additionally act as an assay for drug development studies. In this mini-review, we focus our discussion primarily on whole embryo *Xenopus* models informing cancer biology; the contributions to date and future potential. Insights into tumour immunity, oncogene function, and visualisation of vascular responses during tumour formation have all been achieved with naturally occurring tumours and induced-tumour-like-structures in *Xenopus*. Finally, as we are now entering the era of genetically modified *Xenopus* models, we can harness genome editing techniques to recapitulate human disease through creating embryos with analogous genetic abnormalities. With the speed, versatility and accessibility that epitomise the *Xenopus* system, this new range of pre-clinical *Xenopus* models has great potential to advance our mechanistic understanding of oncogenesis and provide an early *in vivo* model for chemotherapeutic development.

## Introduction

Cancer is a prominent cause of death worldwide and numbers of cases are predicted to increase as populations grow and age (Torre et al., 2016). Oncology research is also expanding in parallel and a wealth of discoveries have provided insight from a social level to intricate details of molecular pathogenesis. These advances would not be possible without the use of research models, but there is increasing pressure to reduce the use of mammalian animals in biomedical research, and there is still a gap for relevant translational models for high-throughput screening for therapeutic development (Naert et al., 2017). The articles in this special issue are testament to the versatility of the *Xenopus* system, and in keeping, the full repertoire of *in vitro* biochemistry, oocytes, explants, embryos, and adult frogs have all provided diverse contributions to cancer research, from understanding biological processes that are deranged in cancer to modelling using a new age of genetically engineered transgenic animals. Here, we summarise the breadth of *Xenopus*’ contribution to cancer research (see also Hardwick and Philpott, 2015), focusing our discussion on the development and application of whole embryos and adult *Xenopus* frogs as *in vivo* models informing cancer biology.

## Parallels Between Development and Disease

The wide-ranging application of *Xenopus* to the field of oncology is built on the striking parallels between tumour pathogenesis and early embryo development. The “hallmarks of cancer” are now widely recognised as abnormal and tumorigenic properties (Hanahan and Weinberg, 2000, 2011), yet they often arise from inappropriate re-activation or de-regulation of normal physiological processes, many of which are instrumental to embryogenesis when precisely executed in space and time (Aiello and Stanger, 2016). Considerable literature now documents the similarities between early development and tumorigenesis in terms of gene expression, proteasome, signalling pathways, and cell behaviours (Ma et al., 2010). A greater understanding of the physiological processes in the embryo, both in terms of molecular components and regulatory mechanisms, may therefore give insight into tumour pathogenesis and potential therapeutic targets, as illustrated below.

**FIGURE 1:**
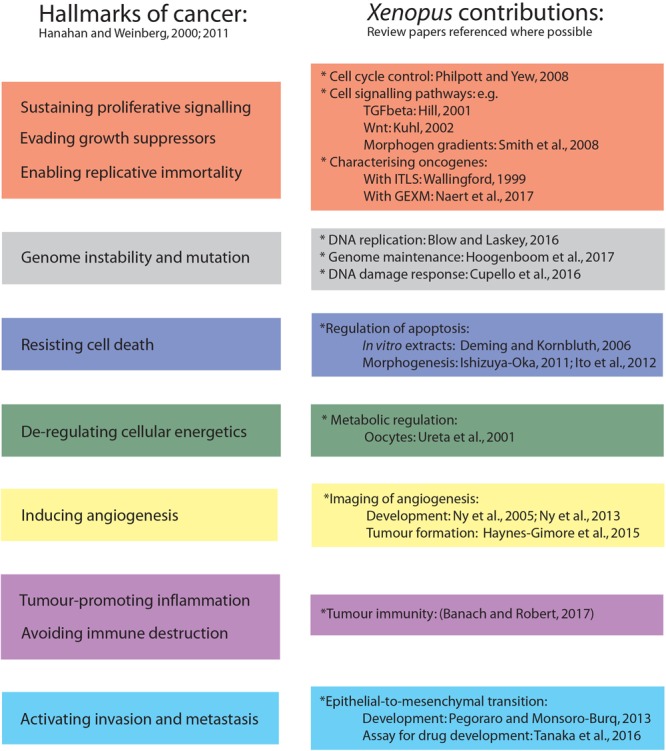
Using the full *Xenopus* repertoire to inform on the hallmarks of cancer. Ten hallmarks of cancer are recognised **(left)** and *Xenopus* studies have informed on each of these aspects **(right)** as described in the main text. Where possible review papers are referenced to direct readers to further discussion of each topic. ITLSs, induced-tumour-like-structures; GEXM, genetically engineered *Xenopus* models.

To summarise the diverse application of the *Xenopus* system to cancer research, Figure 1 illustrates contributions made to understanding each of the cancer hallmarks. This includes the use of *in vitro* extract systems, oocytes and developing *Xenopus* embryos to study fundamental aspects of cell biology such as DNA replication (Blow and Laskey, 2016), genome maintenance (Hoogenboom et al., 2017), DNA damage response (Cupello et al., 2016), cell cycle control (Philpott and Yew, 2008), metabolism (Ureta et al., 2001), and signalling pathways (Hill, 2001; Kuhl, 2002; Smith et al., 2008; Pera et al., 2014). Natural tumour immunity in amphibians has also elucidated immune system interactions (Banach and Robert, 2017), and together with *in vitro* experiments using extracts (Deming and Kornbluth, 2006), morphogenesis of embryos provides a physiological setting for study of apoptosis (Ishizuya-Oka, 2011; Ito et al., 2012). Similarly, key stromal interactions during tumour growth and metastasis have been informed by intra-vital imaging in *Xenopus* tadpoles (Haynes-Gimore et al., 2015) and by the study of developmental epithelial-to-mesenchymal transition (EMT) events (Pegoraro and Monsoro-Burq, 2013) that are key to malignant tumour invasion (Tanaka et al., 2016). Finally, tadpoles and adult frogs contribute to our understanding of genetic influences on tumorigenesis through induced-tumour-like-structures (ITLSs) (Wallingford, 1999) and the use of a new era of genetically engineered *Xenopus* models (GEXM) (Naert et al., 2017).

Increasingly oncologists have harnessed and applied aspects of the developmental biology toolbox, for example utilising lineage tracing technologies to investigate tumour cell origin and evolution (Aiello and Stanger, 2016). Similarly, given that developmental and oncogenic processes often depend on the same underlying pathways, it is perhaps not surprising that embryonic events provide an *in vivo* assay system for discovery and development of new therapeutics targeting these pathways. For example, aberrant Wnt signalling can contribute to all stages of tumorigenesis with a particularly prominent driving role in intestinal cancers, yet it has proved difficult to target therapeutically (Krishnamurthy and Kurzrock, 2018). Axis duplication can be induced in *Xenopus* embryos by injection of RNA encoding β-catenin into a ventral cell of a four cell embryo (Kuhl and Pandur, 2008), and preventing this Wnt-dependent secondary axis is a rapid and efficient screen for potential Wnt inhibitors (Waaler et al., 2011; De Robertis et al., 2013). Furthermore, monitoring developmental events such as tadpole blood and lymphatic formation can be used to screen chemical libraries for anti-angiogenic activity (Kalin et al., 2009), and a transgenic *Xenopus* reporter model now exists with expression of GFP in both blood and lymphatic vasculature under the xFlk1 promoter (Ny et al., 2005, 2013) simplifying such analysis even further. Additionally, gastrulation and neural crest migration critically require transient EMT events, and inhibition of the associated cell movements provides an initial *in vivo* assay to identify compounds that may also inhibit invasion and proliferation of several types of cancer (Tanaka et al., 2016). The large numbers, accessibility and rapid and robust development of embryos have earned *Xenopus* worldwide credibility in developmental biology; these same attributes are now valid for novel pharmacological screening approaches and are likely be utilised further in the future (Schmitt et al., 2014).

## Naturally Occurring *Xenopus* Tumours and Anti-Cancer Defences

Naturally occurring tumours in *Xenopus* are believed to be rare, and potent carcinogens known in mammals have limited ability to induce tumours in amphibians, proving an intriguing insight into mechanisms of relative resistance and/or altered sensitivity to carcinogens. These may include control mechanisms present that regulate adult tissue regeneration, altered self-tolerance post-metamorphosis resulting in cancer cell rejection, ready induction of apoptosis and a reduced sensitivity to DNA damage; reviewed in Ruben et al. (2007). Amphibian skin also secretes a vast array of antibiotic peptides but some of these have inhibitory activity against human cancer cell growth, fuelling interest in defining more of these natural peptide secretions (Li et al., 2016).

### Mechanisms of Tumour Immunity

Inflammation and evading the immune system are two hallmarks of cancer (Hanahan and Weinberg, 2011), and immune-based therapy is already employed in both human (Zhang and Chen, 2018) and veterinary cancer patients (Klingemann, 2018). However, a complex array of tumour and immune cell interactions can have disparate effects, leading to the concept of “immune-editing” of a heterogenous mix of tumour clones (Bui and Schreiber, 2007). While some clones may be completely destroyed by the host immune system, selection pressure can create the persistence of clones with anti-immune defences or reduced immunogenicity, promoting tumour growth (Bui and Schreiber, 2007). Components of the innate and adaptive immune systems are highly conserved between *Xenopus* and mammals; readers are directed to Banach and Robert (2017) for a recent and in-depth review. With the derivation of defined immunogenic *Xenopu*s cancer cell lines (see below), together with in-bred MHC-defined strains of *Xenopus* that permit tumour grafts without rejection, *Xenopus* is a highly valuable model for dissecting the roles of various immune system components; comprehensively reviewed in Goyos and Robert (2009); Robert (2010), and Banach and Robert (2017).

Original reports of highly malignant lymphosarcoma in *Xenopus* were subsequently demonstrated to be an infectious granuloma in response to mycobacterium marinum (Asfari, 1988; Asfari and Thiebaud, 1988), but a range of genuine neoplastic and sometimes metastatic diseases are documented, including hepatoma, teratoma, renal carcinoma, fibrosarcoma, ovarian dysgerminoma, lymphoma, and pancreatic carcinoma (Banach and Robert, 2017). In the 1990s, five thymic lymphomas were reported in genetically different adult frogs, and these have provided five different and now extensively characterised lymphoid cell lines (B3B7, 15/0, 15/40, ff-2, and ff-2.64) (Robert and Cohen, 1998). Features such as high levels of myc expression, aneuploidy and genetic instability indicate their relevance to mammalian cancers, and mixed expression of T and B cell markers indicates resemblance to rare human leukocytic leukaemias (Robert and Cohen, 1998). These different thymic tumour cell lines arise in differing MHC immune backgrounds and display differing behaviours and invasiveness on transplantation, dependent on the immune status of the recipient tadpole or adult (Robert et al., 1994). These transplantation experiments coupled with the ease of genetic manipulation in *Xenopus* have allowed dissection of the mechanisms behind tumour and immune system interactions, including a key role of adult T cells for defence (Robert et al., 1997), and tumour expression of non-classical MHC class1b molecules for escaping immune recognition (Haynes-Gilmore et al., 2014).

### Recent Advances Using Natural Tumour Grafts

Transplantation studies using the aggressive 15/0 tumour cell line and MHC compatible LG6 and LG15 tadpoles have also led to the recent development of a semi-solid tumour model by embedding tumour cells in a collagen matrix prior to subcutaneous engrafting (Haynes-Gimore et al., 2015). By fluorescently labelling tumour cells and infusing intra-cardiac labelled dextran, neoangiogenesis is visualised by intra-vital imaging to reveal a network of convoluted tumour vessels with slow laminar blood flow, recapitulating features of mammalian tumour vasculature. Utilising the natural transparency of *Xenopus* tadpoles, the semi-solid nature of the graft also enables visualisation of collagen rearrangements and infiltrating melanophores, characterising in real-time the tumour interaction with stroma and immune components (Haynes-Gimore et al., 2015). Just as developmental angiogenesis or neural crest formation can provide an *in vivo* screen for inhibitory drugs (see above), this accessible and well-characterised tumour model will surely attract further attention for chemotherapeutic development.

## Induced Tumour-Like-Structures (Itls) as Early *Xenopus* Models of Cancer

Whilst there has been limited success in inducing tumours in *Xenopus* by carcinogens, simple genetic manipulation by RNA injection has been a cornerstone of the *Xenopus* system to study key developmental proteins, and this approach has also yielded early tumour models allowing characterisation of the role of various oncogenes or tumour suppressor proteins. These cancer models further emphasise the closely entwined features of both development and cancer, with developmental factors having influence on tumorigenesis, and the discovery that known oncogenic proteins may originally have a physiological role in development (Wallingford, 1999).

### Developmental Regulators Influencing Oncogenesis

One of the first tumour phenotypes in *Xenopus* was reported through the study of tumour suppressor protein p53 in normal embryo development (Wallingford et al., 1997). Observations in tissue culture experiments and mouse models have long demonstrated the oncogenic effects of mutant p53, in part arising through a loss of cell cycle control and genomic instability. To investigate its role in embryogenesis and tumorigenesis, researchers turned to *Xenopus* where the rapid development of embryos allows analysis without the complicating effects of *de novo* mutations from inherent genomic instability. Human p53 alterations are the most common genetic abnormality in human cancers, often due to dominant-negative effects, and the human protein is biochemically similar to the *Xenopus* homolog, making *Xenopus* a relevant oncological model (Wallingford, 1999). Accordingly, targeted over-expression of dominant-negative human p53 in *Xenopus* embryos inhibits differentiation in multiple germ layers and produces cellular masses of undifferentiated cells with abnormal nuclear morphologies (Wallingford et al., 1997). This essential role of p53 in normal differentiation, beyond its roles in cell cycle control, is now well-documented in aspects of neurogenesis (Hardwick et al., 2014) and supports the notion that certain cancers may arise from a failure of differentiation rather than overt proliferation defects *per se*; a concept also suggested using a *Xenopus* developmental model for paediatric cancer Neuroblastoma (Wylie et al., 2015).

### Oncogenic Regulators Influencing Development

Oncogenes can often directly influence aspects of cell fate and development. For example, over-expression of the viral oncogene polyoma middle T in *Xenopus* animal cap explants results in re-specification of prospective ectoderm to mesoderm, suggesting common signal transduction pathways between early inductive signals and oncogenic stimuli (Whitman and Melton, 1989). This is also supported by investigations in *Xenopus* using dominant inhibitory ras mutants, revealing a role for proto-oncogene p21^ras^ in transduction of FGF and activin signalling in mesoderm induction (Whitman and Melton, 1992). Additionally, the Rel family of transcriptional activators are a diverse group including oncoprotein c-Rel. The *Xenopus* homolog Xrel3 is a distinct rel protein expressed in two phases of early development, and its over-expression induces tumour formation (Yang et al., 1998). It his highly plausible that a protein with a physiological role in regulating the balance between cell proliferation and differentiation may become subverted and contribute to cancer formation, or alternatively there may be a convergence of oncogenic stimuli on the same developmental signalling pathways.

### Characterising Oncogenes With Induced Tumour Models

Consistent with this theme of aberrant use of signalling pathways in oncogenesis, a *Xenopus* model of human basal cell carcinoma (BCC) has revealed the importance of Gli1 as a target and mediator of sonic hedgehog (Shh) signalling in BCC development. Over-expression of Gli1 in *Xenopus* embryos results in focal epidermal lesions with marker expression resembling that of human BCC and highlighting Gli1 as a potential early biomarker for diagnosis of BCC (Dahmane et al., 1997). Thus, ITLSs have been produced by over-expression of a range of proteins relevant to human cancers, and these tumours demonstrate disorganised and undifferentiated cells, high mitotic indicies, stromal interactions and neovascularisation (Chernet and Levin, 2013).

These same *Xenopus* models have also been used to extensively explore membrane depolarisation as a bioelectric marker predictive of ITLS foci (Chernet and Levin, 2013). Transmembrane potential itself may be an oncogenic driver and depolarisation of native neural crest cells can non-cell autonomously induce neoplastic changes in surrounding melanocytes (Blackiston et al., 2011; Lobikin et al., 2012). Similarly, formation of ITLS can be suppressed by hyperpolarisation of tumour or surrounding cells, suggested to be mediated through enhanced tumour uptake of butyrate with HDAC inhibitory properties, resulting in reduced cellular proliferation (Chernet and Levin, 2013, 2014). For further discussion (see Levin et al., 2017; Silver and Nelson, 2018).

## Genetically Engineered *Xenopus* Models (Gexm)

From the preceding discussion, *Xenopus* models have already assisted with investigation of both cancer pathogenesis and therapeutic development. These models are being expanded to a new dimension by using genome editing technology with TALENs or CRISPR/Cas9; methodology discussed in Naert et al. (2017). Genetically engineered mouse models are well-established in the Oncology toolbox, but aquatic models like zebrafish and *Xenopus* offer extra-uterine development of large embryos and simple injection set-ups for delivery of targeted nucleases, also usually lacking inherent complications of highly inbred genetic backgrounds (Naert et al., 2017). In addition to rapid development, high embryo number, and detailed fate maps for tissue-specific targeting, *Xenopus* also presents several advantages over zebrafish, such as reduced evolutionary distance to humans. Moreover, *Xenopus tropicalis* has a true diploid genome with substantial shared synteny with the human genome; in zebrafish whole genome duplication leads to redundancy and complications identifying human orthologs (Naert et al., 2017).

The first GEXM was established to phenocopy Familial Adenomatous Polyposis (FAP) by TALEN-mediated targeting of the *apc* gene in *Xenopus tropicalis*, replicating the human frame-shifting mutations and complementing models such as the Apc^min^ mouse and ENU-induced *apc* zebrafish mutant (Van Nieuwenhuysen et al., 2015). FAP is an autosomal dominant disease due to truncating mutations of the *apc* gene, resulting in 100s to 1000s of adenomatous polyps in the colon, potentially progressing to adenocarcinoma and sometimes accompanied by extra-colonic manifestations. Although F0 tadpoles do not develop genuine intestinal adenomas, they do display abnormal histological architecture of the intestine and the lack of adenomas may be explained by the distinct *Xenopus* intestinal folding pattern that allows proliferating cells to spread rather than form polyps (Van Nieuwenhuysen et al., 2015). Additionally, retinal hyperplasia and external tumours such as subcutaneous desmoid tumours resemble extra-colonic disease reported in humans, each displaying increased Wnt signalling and only mutant *apc* alleles. Thus, the *Xenopus apc* model provides high penetrance and rapid and reproducible tumour formation that is comparable to the human disease (Van Nieuwenhuysen et al., 2015).

A second model by the same group used CRISPR/Cas9-mediated knockout of *rb1* and *rbl1* genes in *Xenopus tropicalis* to phenocopy Retinoblastoma, a paediatric tumour of the developing retina (Naert et al., 2016). While mouse models exist, they show variable latency to tumour development and rely on conditional deletion as complete knock-out is embryonic lethal, while a zebrafish model relies on orthotopic retinoblastoma transplantation. As in mice, *Xenopus* tumours require inactivation of both *rb1* and *rbl1* genes, achieved by co-injection of independent pairs of guide RNAs and editing by CRISPR-Cas9; these mosaic double knock-out tadpoles develop a rapid and penetrant retinoblastoma in as little as 35 days, with histopathology and disease progression conserved with the human tumour (Naert et al., 2016). Efficiency of genome editing is reported at 25–30% for each locus, thus neoplasias develop in F0 mosaic mutants without high genome editing efficiencies, and given that an entire experiment can be conducted within 3 months, this technology using the *Xenopus* system has huge potential for future application (Naert et al., 2016).

## Future Perspectives

Building on the extensive history of *Xenopus* in exploring fundamental cell and developmental biology, we are now entering a new era where modern genome editing technology is being combined with all the classical attributes of the *Xenopus* system to generate clinically relevant cancer models that are rapid, penetrant and highly suited to high-throughput screening. This is an important step allowing reduction of the number of mammals in pre-clinical research, and provides a range of platforms for therapeutic development, either using developmental events to screen for drugs targeting the same signalling pathways or as relevant *in vivo* tumour models. Furthermore, the aquatic nature of *Xenopus* permits fast and efficient preclinical screening of water-soluble compounds (Naert et al., 2017). There is also potential for characterising disease modifying genes by multiplexed biallelic targeting in *Xenopus* and proof of principle is already shown for dual target genes with TALENs (Naert et al., 2017) and triplex gene editing with CRISPR (Naert et al., 2016). Next steps will involve deletion of large chromosomal regions or replicating chromosomal translocations and relocations (Naert et al., 2017). Thus, *Xenopus* has rightfully earned a place in the Oncologist’s toolbox and is likely to achieve even more prominence as the unique advantages of the *Xenopus* system become widely acknowledged in the oncology field.

## Author Contributions

LH wrote the manuscript and prepared the figure. AP reviewed and edited the manuscript.

## Conflict of Interest Statement

The authors declare that the research was conducted in the absence of any commercial or financial relationships that could be construed as a potential conflict of interest.
